# A Heterozygous Mutation in Cardiac Troponin T Promotes Ca^2+^ Dysregulation and Adult Cardiomyopathy in Zebrafish

**DOI:** 10.3390/jcdd8040046

**Published:** 2021-04-20

**Authors:** Sarah M. Kamel, Charlotte D. Koopman, Fabian Kruse, Sven Willekers, Sonja Chocron, Jeroen Bakkers

**Affiliations:** 1 Hubrecht Institute-KNAW, University Medical Center Utrecht, 3584 CT Utrecht, The Netherlands; s.kamel@hubrecht.eu (S.M.K.); l.koopman@hubrecht.eu (C.D.K.); Kruse.fabian@outlook.com (F.K.); s.willekers@hubrecht.eu (S.W.); s.chocron@hubrecht.eu (S.C.); 2 Department of Medical Physiology, Division of Heart & Lungs, University Medical Center Utrecht, 3584 CM Utrecht, The Netherlands; 3 Department of Pediatric Cardiology, Division of Pediatrics, University Medical Center Utrecht, 3584 CM Utrecht, The Netherlands

**Keywords:** cardiac Troponin T, cardiomyopathy, zebrafish, structural remodeling, calcium dysregulation, contractility defects, CRISPR/Cas9

## Abstract

Cardiomyopathies are a group of heterogeneous diseases that affect the muscles of the heart, leading to early morbidity and mortality in young and adults. Genetic forms of cardiomyopathy are caused predominantly by mutations in structural components of the cardiomyocyte sarcomeres, the contractile units of the heart, which includes cardiac Troponin T (TnT). Here, we generated mutations with CRISPR/Cas9 technology in the zebrafish *tnnt2a* gene, encoding cardiac TnT, at a mutational “hotspot” site to establish a zebrafish model for genetic cardiomyopathies. We found that a heterozygous *tnnt2a* mutation deleting Arginine at position 94 and Lysine at position 95 of TnT causes progressive cardiac structural changes resulting in heart failure. The cardiac remodeling is presented by an enlarged atrium, decreased ventricle size, increased myocardial stress as well as increased fibrosis. As early as five days post fertilization, larvae carrying the TnT RK94del mutation display diastolic dysfunction and impaired calcium dynamics related to increased Ca^2+^ sensitivity. In conclusion, adult zebrafish with a heterozygous TnT-RK94del mutation develop cardiomyopathy as seen in patients with TnT mutations and therefore represent a promising model to study disease mechanisms and to screen for putative therapeutic compounds.

## 1. Introduction

Cardiomyopathies are a heterogeneous group of diseases that affect the myocardium, causing sudden cardiac death and early mortality in children and adults [1]. Patients within the same family can range from being asymptomatic to having mild-severe symptoms [2]. There are several types of cardiomyopathies that are categorized based on their morphological and pathophysiological differences. These include hypertrophic cardiomyopathy (HCM), dilated cardiomyopathy (DCM), and restrictive cardiomyopathy (RCM) [3]. Initially, it was thought that these individual diseases have distinctive features where HCM has a thickened left ventricle wall and septum, while DCM causes dilation and thinning of the left ventricle wall, and RCM promotes stiffening of the ventricle walls and restrictive filling. However, in recent years, it is becoming more prominent that these cardiomyopathy types can come as a spectrum of disease with overlapping features [4,5]. These features can include the progression of fibrosis, changes in the ventricle wall structure, disruption in the contractility and intracellular calcium (Ca^2+^), and dysregulation in the electrical functions of the heart. Although these diseases can have various underlying factors, many progress as a result of mutations in proteins of the sarcomere, the contractile unit of the striated muscles. Hence, they are referred to as sarcomeric cardiomyopathies.

Sarcomeres are composed of thick and thin filaments that slide along each other during muscle contraction. In the thin filaments, cardiac Troponin T (TnT) forms a troponin complex with Troponin I (TnI) and Troponin C (TnC) to interact with Tropomyosin (Tm) coiled-coil dimers. These components closely interact with actin molecules to regulate muscle contraction [2]. The thick filaments of the sarcomeres consist of myosin heavy chains and four myosin light chain molecules. These myosin light chains provide mechanical support to the heavy chain and regulate the interaction of the myosin heads with actin, which eventually affects the force and velocity of contraction [2,6]. The principle of muscle contraction relies on the sliding of actin past myosin to create muscle tension, which is controlled by intracellular [Ca^2+^]. When an action potential is triggered, voltage-sensitive L-type Ca^2+^ channels (LTCCs) are activated, leading to Ca^2+^ ion flow into the cytosol of the cardiomyocytes. Ca^2+^ binds the contractile elements of the cell at TnC to induce allosteric conformational changes in TnI and TnT, which are transmitted to Tm. This conformational change results in force development by actin and myosin, and contraction occurs due to shortening of the sarcomeres [6]. During relaxation, Ca^2+^ is taken up by the sarcoplasmic reticulum (SR) and shuttled out of the cell through Na^+^/Ca^2+^ exchanger (NCX) causing relaxation of the cardiomyocyte [7]. Ca^2+^ signaling and handling are affected in cardiomyopathies due to mutations in the genes encoding the myofilament proteins [8].

The *TNNT2* gene encodes the thin filament contractile protein TnT [6]. The principle isoform of *TNNT2* in the adult human heart encodes 288 amino acids and consists of three structural regions (from N to C terminus): (1) the hypervariable region (residues 1–79) that does not interact with any proteins, (2) the TNT1 region (residues 80–180) which interacts and overlaps with Tm dimer, and (3) the TNT2 region (residues 181–288) that interacts with Tm and the globular end of the troponin complex (TnI and TnC) [8]. Clinical studies on families with mutations in *TNNT2* show missense mutations that mostly occur within the TNT1 region (residues 92–144), leading to the progression of cardiomyopathies [9]. This region, in fact, is known as the mutational “hotspot” as mutations that occur here cause changes in the binding affinity of TnT to Tm. Changes in biding affinity lead to alterations in Ca^2+^ levels, contractility, and the progression of cardiomyopathies [9]. These mutations result in variable symptoms that depend on mutation penetrance, coinciding with the previously mentioned common features of H/D/RCM [10,11,12,13,14,15].

During the past decades, the zebrafish (*Danio rerio*) has emerged as a favorable model to study cardiovascular development and disease. Embryos that lack blood circulation can still survive by relying on passive diffusion of oxygen to the tissues; this allows the embryos to survive the initial stages of embryonic development to study any cardiovascular defects that might arise [16,17,18,19]. A mutant named *silent heart* (*sih*) and affecting the *tnnt2a* gene was one of the first embryonic cardiomyopathy models established. Homozygous *sih/tnnt2a* mutant embryos lack cardiac contraction, develop pericardial edema, and display defects in thin filament and sarcomere assembly [20].

Thus far, only embryonic studies of homozygous loss-of-function mutants of *tnnt2a* have been conducted. As cardiomyopathies arise in young adults with heterozygous mutations in sarcomeric genes including *TNNT2*, we were intrigued to find out whether heterozygous mutations in the zebrafish *tnnt2a* gene would result in cardiomyopathies in post-embryonic stages, especially if they are targeting the mutational “hotspot” of *TNNT2*. Here, we used CRISPR/Cas9 technology to generate an out-of-frame and an in-frame deletion of two amino acids in exon 9 of *tnnt2a.* While homozygous *tnnt2a* out-of-frame deletions are embryonically lethal and result in the *sih* phenotype, fish heterozygous for this mutation display no cardiac phenotypes. Homozygous *tnnt2a* in-frame deletions are also embryonically lethal; however, our findings indicate that heterozygous zebrafish develop a severe form of cardiomyopathy at the juvenile-adult stages of development. We show that these fish undergo cardiac remodeling, which is accompanied by an increase in myocardial stress and fibrosis. Furthermore, this structural remodeling is preceded by functional cardiac defects that are typical for human familial cardiomyopathies.

## 2. Materials and Methods

### 2.1. Zebrafish Husbandry

Fish used in this study were housed under standard conditions as described previously [21]. All experiments were conducted in accordance with the ethical guidelines and approved by the local ethics committee of the Royal Dutch Academy of Sciences (KNAW).

### 2.2. Generation of Mutant Lines

*Tnnt2a* lines were generated using CRISPR/Cas9 genome editing technology. Open reading frame of *tnnt2a* was chosen using CHOPCHOP (http://chopchop.cbu.uib.no/, accessed on 1 May 2014 [22]). sgRNA template was purchased from Integrated DNA Technologies (IDT) as standard desalted same-day oligo, and synthesis was carried out using MEGAshortscript T7 kit (Ambion). Purification of the in vitro synthesized mRNA was performed using RNeasy Mini Kit (Qiagen). sgRNA and Cas9-encoding mRNA were co-injected into one-cell stage wild-type (TL) zebrafish embryos at a concentration of 12.5 ng/µL sgRNA and 150 ng/µL Cas9-encoding mRNA. Injected embryos were grown to adulthood to identify F0 founder fish with indel mutations and screen for germline transmission. Each putative founder adult zebrafish was crossed with a wild-type adult fish (F1). Details of sequencing primers and sgRNA sequences can be found in Figure S1. Sequencing analysis of the targeted region revealed mutation carrier fish with either 5 or 6 nucleotide deletion (referred to as *tnnt2a*^+/hu11260^ and *tnnt2a*^+/RK94del^).

### 2.3. Adult Zebrafish Heart Isolations and Preparation

For paraffin sections, adult zebrafish hearts were dissected and fixed in 4% paraformaldehyde (dissolved in phosphate buffer containing 4% sucrose) at 4 °C overnight, washed twice in PBS, dehydrated in EtOH, and embedded in paraffin. Serial sections were made using a microtome (Leica RM2035, Leica, Wetzlar, Germany). Different section thicknesses were used, depending on the size/age of the sample: 10 μm for adult hearts, 8 μm for juvenile hearts, and 6 μm for embryos. For cryosections, zebrafish hearts were extracted and fixed in 4% paraformaldehyde (in phosphate buffer) for 4 h at room temperature (RT). Three washes of 30 min were performed using 4% sucrose (in phosphate buffer) followed by an overnight incubation at 4 °C in 30% sucrose (in phosphate buffer). Hearts were embedded in tissue-freezing medium (Leica, Lot# 03811456), frozen on dry ice, and kept at −80 °C. Cryo-sectioning using Cryostar NK70 (Thermo Scientific, Breda, The Netherlands) was performed to obtain 10 μm thin sections. Images of extracted whole hearts were acquired using a Leica M165 FC stereomicroscope.

### 2.4. Adult Zebrafish Heart Staining

In situ hybridization (ISH) was performed on paraffin sections as described previously [23], with the exception that the hybridization buffer did not contain heparin and yeast total RNA. *vmhcl, amhc, nppb, tnnt2a*, and *col6a1* probes were previously generated [24]. Primers for the *postnb* probe generation were forward primer AGAGGTTCTGGACAGGCTCA and reverse primer AAGGCACCATTTTTCACCAG. Haematoxylin and eosin (H&E) staining and acid fuchsin-orange staining (AFOG) were performed on paraffin and cryosections, respectively, in accordance with standard laboratory protocols. Imaging of stained sections was performed using Leica DM4000 B LED upright automated microscope and Olympus Slideview VS200 digital slide scanner.

### 2.5. High-Speed Brightfield Imaging

Embryos were placed in 200 µM 1-phenyl-2-thiourea (PTU) 20–24 hpf to prevent pigmentation. At 5 dpf, embryos were embedded in 0.3% agarose prepared in E3 medium containing 16 mg/mL MS-222. Recordings were performed at 150 frames per second (fps) using a high-speed inverted light microscope at 28 °C. Heart rate measurements and contractility parameters were analyzed using ImageJ (USA National Institutes of Health, Bethesda, MD, USA). Contraction time, relaxation time, and systolic duration were analyzed by going through the recording frame by frame manually and observing the moments when (1) the ventricular wall moved inward, and the ventricle started expelling blood (start of contraction), (2) the ventricle wall moved back outward (start of relaxation), and (3) the ventricle wall was back at its most dilated position (maximum relaxation). After repeating the process 3 times for each heart, values were averaged, and contractile parameters were calculated. The contraction and relaxation time were corrected for heart rate (HR) by using the formula: time/√(beat to beat interval). The systolic fraction was calculated from the systolic time by using: (systolic time × R)/60.000. The hemodynamic parameters such as surface area and volumes were analyzed using ImageJ by drawing an ellipse on top of the ventricle at end-diastole and end-systole. Six ellipses were analyzed per heart: 3 at diastole and 3 at systole. Values were averaged. ImageJ provided the values for the minor and major axis of each ellipse. End-diastolic and end-systolic volume (EDV/ESV) were calculated by: (1/6) × (π) × (major axis) × (minor axis^2^). Stroke volume (SV) by: EDV-ESV. Ejection fraction (EF) by: SV/EDV. Cardiac output (CO) by: SV × Heart rate. Surface area was calculated using the following formula: (0.5 × major axis) × (0.5 × minor axis) × π. Fractional area change (FAC) was calculated by: (area diastole − area systole)/(area diastole).

### 2.6. High-Speed Fluorescence Imaging

*tnnt2a*^+/RK94del^ fish were crossed to *tg* (*myl7*:Gal4FF; *UAS*:GCaMP6f) fish [25] to obtain *tnnt2a* fish with a genetically encoded cardiac Ca^2+^ sensor. Embryos were placed in 200 µM PTU after 20–24 hpf to keep them transparent. At 5 dpf, embryos’ heart contraction was stopped by incubation with E3-MS222-PAB-BDM mixture (PAB: Para-amino blebbistatin from Optopharma, Budapest, Hungary, Cat# DR-Am-89 at 5 µM; BDM: 2,3-Butanedione monoxime from Sigma-Aldrich, St Louis, MO, USA, Cat# B0753 at 100 µM) for 1 h at 28 °C. Embryos were embedded in 0.3% agarose prepared in E3 medium containing 16 mg/mL MS222-100 µM BDM and placed in a heated (28 °C) recording chamber bath containing 100 µM BDM in E3. Recordings were performed using a custom-built upright widefield microscope (Cairn Research, Faversham, UK) equipped with a 20 × 1.0 NA objective (Olympus XLUMPLFLN20X W, Olympus, Tokyo, Japan). Blue LED excitation light (470 nm) was filtered using a 470/40 nm filter (Chroma ET470/40x, Chroma, Bellows Falls, VT, USA) and reflected toward the objective using a 515 nm dichroic mirror (Chroma T515lp). Emitted fluorescence was filtered by a 514 long-pass filter (Semrock LP02-514RU, Semrock, New York, NY, USA), and images were projected on a high-speed camera (Andor Zyla 4.2 plus sCMOS, Andor, Belfast, Northern Ireland ). Recordings were performed at 100 fps, for 1000 frames. Parameters such as heart rate, Ca^2+^ transient amplitude, peak Ca^2+^ level, diastolic Ca^2+^ level, upstroke time, and recovery time were recorded. Recordings were analyzed using ImageJ and Matlab (Version R2015a, Mathworks, Natick, MA, USA).

### 2.7. Statistical Analysis

Statistical analysis and drawing of graphs and plots were carried out in GraphPad Prism (version 7 for Windows, GraphPad Software, San Diego, CA, USA). Differences between two groups were analyzed using the unpaired Student’s *t*-test, with the exception of the Kaplan–Meier curve, where Log-rank (Mantel–Cox) test was used. All data are presented as mean ± SEM, and *p* < 0.05 was considered significant. * *p* ≤ 0.05, ** *p* ≤ 0.01, *** *p* ≤ 0.001, **** *p* ≤ 0.0001, n.s. *p* > 0.05. N denotes the number of fish used per dataset.

## 3. Results

### 3.1. Targeting Exon 9 of tnnt2a via CRISPR/Cas9 Promotes Changes in Heart Morphology of Adult Zebrafish

**Figure 1:**
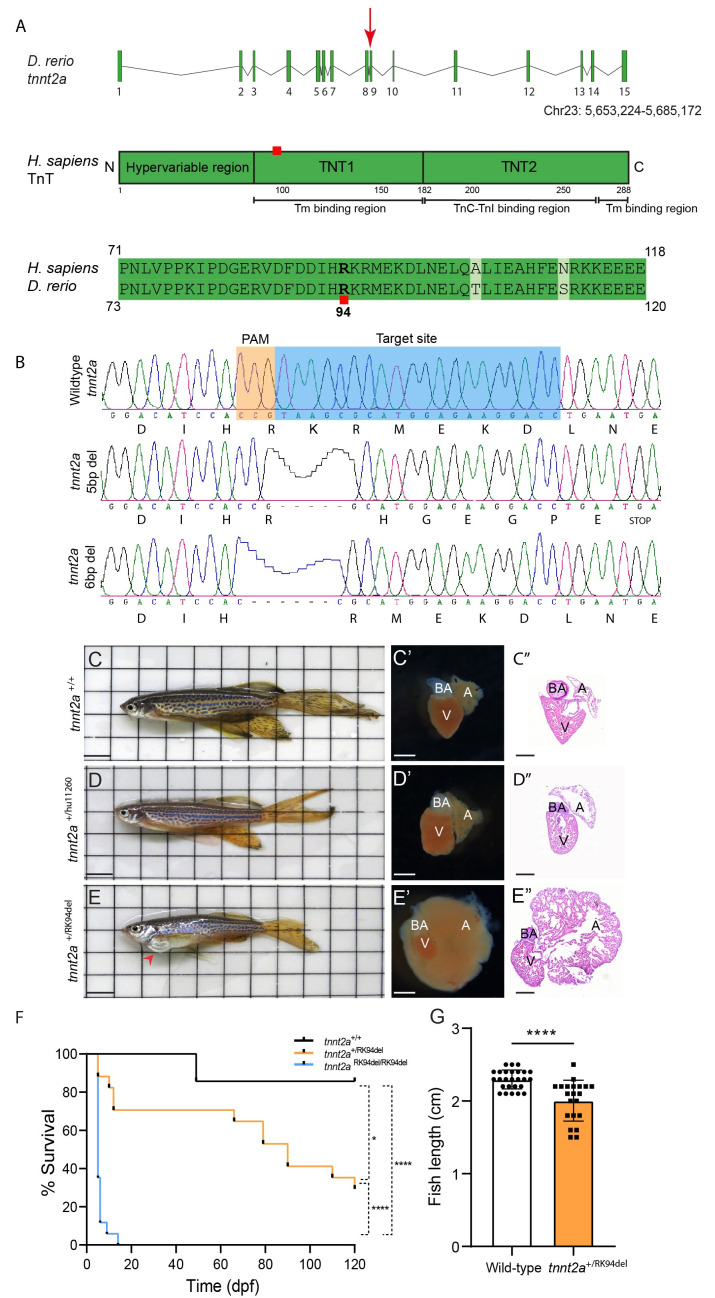
CRISPR/Cas9-induced mutation in *tnnt2a* promotes the formation of cardiomyopathy in adult zebrafish. (**A**) Illustration of *tnnt2a* exons, introns, and chromosome location. Red arrowhead indicates the CRISPR/Cas9 mutation location. The cardiac Troponin T (TnT) human protein is schematically depicted, showing the different regions (hypervariable region, TNT1, and TNT2) that interact with tropomyosin I binding region and Troponin C and I (TnC-TnI) binding region, spanning 288 amino acids. The Arginine mutation site is highlighted with a red square, located on position 94 in zebrafish (*D. rerio*), which corresponds to the Arginine amino acid 92 in humans (*H. sapiens*). (**B**) Two different mutations targeting exon 9 of *tnnt2a* were identified, either causing a 5 bp frameshift deletion and a truncated form of the protein or a 6 bp in-frame deletion causing a mutated form of the protein. The target sequence for CRISPR/Cas9 is shown in blue and the PAM sequence in orange. The amino acid sequences show the effect of the mutations on the protein. (**C**–**E**) Images of 4-month-old adult zebrafish with the genotypes *tnnt2a*^+/+^, *tnnt2a*^+/hu11260^, or *tnnt2a*^+/RK94del^, respectively. Scale bar of 0.5 cm. (**C**’–**E**’) Extracted hearts of the respective fish. Scale bar of 100 µm. (**C**”–**E**”) Haematoxylin and Eosin (H&E) stainings of heart sections from *tnnt2a*^+/+^, *tnnt2a^+/hu11260^,* or *tnnt2a^+/RK94del^*, respectively. Images were taken at a magnification of 20×, and scale bars are 100 µm. A: atrium, V: ventricle, BA: bulbus arteriosus. (**F**) Kaplan–Meier curve, displaying the survival of *tnnt2a*^+/+^, *tnnt2a*^+/RK94del^, and *tnnt2a*^RK94del/RK94del^ fish during the first 120 days of development (*n* = 7, 17, and 17, respectively). Statistics: Log-rank (Mantel–Cox) test, mean ± SEM, * *p* ≤ 0.05, **** *p* ≤ 0.0001. (**G**) Measurement of the length of adult fish from *tnnt2a*^+/+^ and *tnnt2a* ^+/RK94del^. Statistics: mean ± SEM, **** *p* ≤ 0.0001, *tnnt2a*^+/+^ *n* = 26, *tnnt2a* ^+/RK94del^ *n* = 21, unpaired Students *t*-test.

Zebrafish *tnnt2a* is located on chromosome 23 and consists of 15 exons. Exon 9 encodes for a highly conserved region within the Tm binding region which is often mutated in patients with familial cardiomyopathy, including the Arginine 92 site (corresponding to Arginine 94 in zebrafish) [9] (Figure 1A). We therefore designed a guide RNA to target this region for mutagenesis with CRISPR/Cas9. From this, we recovered two F1 lines with different mutations. In one of the lines (hu11260), we identified a deletion of five nucleotides causing a frameshift followed by a stop codon resulting in a truncated protein lacking most of the Tm binding region and the entire TnC-TnI binding region. In the second line (hu11320), we identified an in-frame deletion of six nucleotides resulting in the deletion of an Arginine at position 94 and a Lysine at position 95, which hereafter is referred to as *tnnt2a*^+/RK94del^ (Figure 1A,B).

Homozygous *tnnt2a*^hu11260/hu11260^ embryos at two days post fertilization (dpf) showed normal embryo morphology but lacking heart contraction, which resembles the previously reported *tnnt2a* loss-of-function mutation [20]. Heterozygous *tnnt2a*^+/hu11260^ zebrafish were viable with no apparent phenotypic changes, including normal heart rate at 5 dpf, and looked similar to their wild-type siblings in embryonic and adult stages (Figure 1C,D and Figure S2). Moreover, extracted hearts of adult *tnnt2a*^+/hu11260^ zebrafish appeared normal, with no visible morphological changes (Figure 1C′,C″,D′,D″). Homozygous *tnnt2a*^RK94del/RK94del^ embryos were suffering from defects after heart looping, evident by the lack of heart chamber ballooning, pericardial edema, and weak heart contraction. Interestingly, also heterozygous *tnnt2a*^+/RK94del^ embryos showed heart defects with variable penetrance. Survival count revealed that *tnnt2a*^RK94del/RK94del^ embryos die within the first two weeks (Figure 1F, Figure S3). Strikingly, 70% of *tnnt2a*^+/RK94del^ zebrafish survive the first two weeks but suffer from heart failure at ages ranging from 10 to 20 weeks post-fertilization (wpf) and have a higher susceptibility for bacterial infection. Only around 30% of *tnnt2a*^+/RK94del^ survive the first four months of life (120 dpf) (Figure 1F). Females are noticed to have a decline in their egg production as early as 13 wpf. From 10 wpf onward, *tnnt2a*^+/RK94del^ mutants develop protruding hearts combined with reduced body length (Figure 1G). Hearts extracted from 12-weeks-old *tnnt2a*^+/RK94del^ zebrafish were enlarged, and it was often hard to distinguish the atrium and the ventricle (Figure 1E–E″). This heart remodeling was observed in 100% of the *tnnt2a*^+/RK94del^ fish (*n* = 78) with variable degrees of severity, compared to no remodeling in their wild-type siblings (*n* = 109). In addition, the phenotype was consistent in more than 15 generations in which the *tnnt2a*^+/RK94del^ zebrafish were outcrossed with wild-type TL fish, indicating a strong genotype/phenotype correlation. Fish with severely remodeled hearts suffered from breathing difficulties, evident by swimming at the surface gasping for air. These fish also showed a significant decrease in size and length, compared to their wild-type siblings (Figure 1E,G). In conclusion, by targeting exon9 in *tnnt2a,* we generated different alleles that display very different phenotypes. While the 5 bp out-of-frame deletion in *tnnt2a* acts as a recessive mutation and does not cause disease in heterozygous fish, the 6 bp deletion that removes Arg94 and Lys95 is dominant and causes phenotypes in heterozygous fish, including heart failure in adults.

### 3.2. Heart Morphological Changes in tnnt2a^+/RK94del^ Can Be Observed at Early Larval Stages of Development

**Figure 2:**
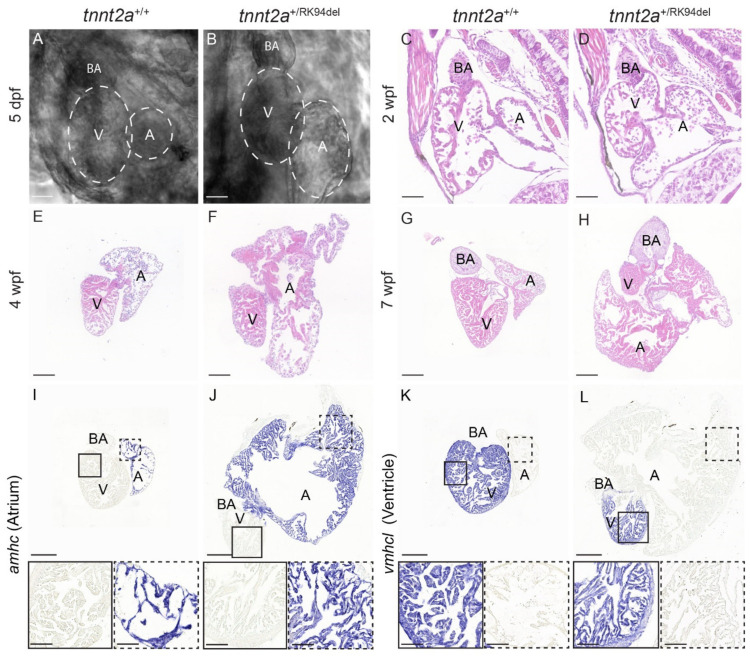
Morphological changes in *tnnt2a*^+/RK94del^ observed during different stages of development. (**A**,**B**) Images of 5 dpf embryonic hearts for *tnnt2a*^+/+^ and *tnnt2a*^+/RK94del^. (**C**–**H**) H&E staining of heart sections of *tnnt2a*^+/+^ and *tnnt2a*^+/RK94del^ at (**C**,**D**) 2 weeks post-fertilization, (**E**,**F**) 4 weeks post-fertilization, and (**G**,**H**) 7 weeks post-fertilization. (**I**–**L**) In situ hybridization on adult heart sections of *tnnt2a*^+/+^ and *tnnt2a*^+/RK94del^ stained for (**I**,**J**) *amhc*, a marker of atrial cells, and (**K**,**L**) *vmhcl*, a marker for adult zebrafish ventricular cells. Images were taken at a magnification of 20×. Scale bars are 100 µm (**A**–**F**), 50 µm (**G**–**I**), and 200 µm for whole-heart tile scans (**I**–**L**) with 50 µm for zoom-in regions. A: atrium, V: ventricle, BA: bulbus arteriosus.

**Figure 3:**
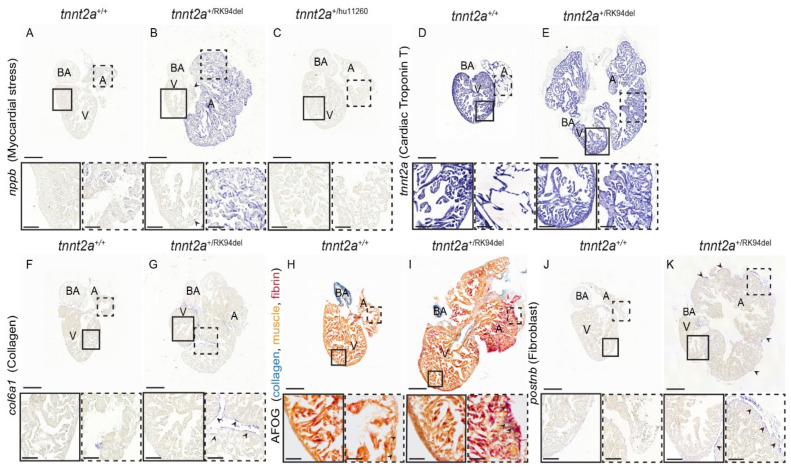
Hearts of adult *tnnt2a*^+/RK94del^ display increased myocardial stress and fibrosis. (**A**–**C**) In situ hybridization on 4-month-old adult heart sections of *tnnt2a*^+/+^, *tnnt2a*^+/RK94del^, and *tnnt2a*^+/hu11260^ stained for *nppb*, a marker of myocardial stress. In situ hybridization on adult heart sections of *tnnt2a*^+/+^ and *tnnt2a*^+/RK94del^ stained for (**D**,**E**) *tnnt2a***,** to mark cardiac Troponin T expression, and (**F**,**G**) *col6a1*, which marks collagen. (**H**,**I**) AFOG staining, which marks collagen in blue, muscle in orange, and fibrin in red of *tnnt2a*^+/+^ and *tnnt2a*^+/RK94del^. (**J**,**K**) In situ hybridization on adult heart sections of *tnnt2a*^+/+^ and *tnnt2a*^+/RK94del^ stained for *postnb*, to mark fibroblasts. Images were taken at a magnification of 20×, except for (**H**,**I**) where magnification was at 60×. Scale bars are 200 µm for whole-heart tile scans, 50 µm for zoom-in regions, and 20 µm for zoom-in regions of H, I. A: atrium, V: ventricle, BA: bulbus arteriosus.

As *tnnt2a*^+/RK94del^ fish developed phenotypes such as a protruding heart and altered swimming behavior at 10 weeks of age, we wanted to investigate whether morphological differences in the heart occurred and when these started to develop. To achieve this, live imaging on 5 dpf larvae was performed, as well as histological analysis at three different time points being 2, 4, and 7 wpf. Surprisingly, changes were observed at 5 dpf in the atrium. The atrium appeared larger in 20% of *tnnt2a*^+/RK94del^ larvae, while the ventricle seemed to be unaffected (*n* = 24) (Figure 2A,B). Histological analysis confirmed this finding and showed that by 2 wpf, the size of the atrium was slightly increased in all *tnnt2a*^+/RK94del^ fish, while the size of the ventricle seemed unaffected (*n* = 6) (Figure 2C,D). Remodeling becomes more apparent at 4 wpf, when the atrium of *tnnt2a*^+/RK94del^ fish has enlarged further, and the ventricle appears slightly smaller in comparison to the wild-type siblings (*n*= 6) (Figure 2E,F). By 7 wpf, the size of the atrium in *tnnt2a*^+/RK94del^ fish is grossly enlarged, and a clear decrease in the size of the ventricle is now apparent (*n* = 6) (Figure 2G,H). To better distinguish the atrium from the ventricle histologically, we analyzed the expression of *atrial myosin heavy chain* (*amhc*) and *ventricle myosin heavy chain-like* (*vmhcl*) via mRNA in situ hybridization. Both *amhc* and *vmhcl* are expressed at comparable levels in hearts of adult *tnnt2a*^+/+^ and *tnnt2a*^+/RK94del^ zebrafish, but the atrium of *tnnt2a*^+/RK94del^ zebrafish is much larger and contains a thicker outer layer compared to wild-type controls (*n* = 6) (Figure 3I,J). The nuclei in the atria of the *tnnt2a*^+/RK94del^ appear abundant but similarly spaced as in wild-types, suggesting that hyperplasia (an increase in cell number) rather than hypertrophy (an increase in cell size) causes the enlargement of the atrium. The ventricle of *tnnt2a*^+/RK94del^ fish appears to be morphologically similar to wild-type but is strongly reduced in size (*n* = 6) (Figure 3K,L). Together, these results demonstrate that cardiac remodeling in *tnnt2a*^+/RK94del^ fish starts as early as the larval stage and is characterized by a progressive enlargement of the atrium and a reduction of the ventricle.

### 3.3. Heart of Adult tnnt2a^+/RK94del^ Displays Increased Myocardial Stress

As we observed a strong remodeling in *tnnt2a*^+/RK94del^ hearts, we wondered whether genes that are upregulated in failing mammalian hearts, such as natriuretic peptides [26], are also upregulated in *tnnt2a*^+/RK94del^ hearts. Indeed, we detected a higher expression of *Natriuretic peptide type B* (*nppb*) in the atrium of the *tnnt2a*^+/RK94del^ hearts and some sparse expression in the outer edges of the ventricle (*n* = 6) (Figure 3A,B, arrowheads). As previous results indicate that the atrium is more severely affected than the ventricle, we investigated whether *tnnt2a* is differentially expressed between the two heart chambers. In situ hybridization revealed no difference in *tnnt2a* expression between the atrium and ventricle of wild-type and *tnnt2a*^+/RK94del^ hearts (*n* = 6) (Figure 3D,E). As *tnnt2a*^+/RK94del^ hearts express only one wild-type copy of the *tnnt2a* gene, we wondered whether the loss of one *tnnt2a* wild-type copy is sufficient to induce the observed cardiac remodeling. However, as the hearts of *tnnt2a*^+/hu11260^ fish, which also have only one wild-type *tnnt2a* copy, show normal morphology (*n* = 6) and lack upregulation of *nppb* (Figure 3C), remodeling has to be due to the RK94del mutation. Based on these findings, we concluded that *tnnt2a*^+/RK94del^ mutants develop evident morphological changes of the heart, characterized by an enlarged atrium and a smaller ventricle. These changes are accompanied by myocardial stress throughout the mutant heart.

### 3.4. Detection of Increased Collagen and Fibrin Deposition in Atrium of Adult tnnt2a^+/RK94del^ Hearts

Myocardial stress and remodeling are often accompanied by increased fibrosis. To investigate whether this also occurred in *tnnt2a*^+/RK94del^ hearts, *col6a1* expression was analyzed. *col6a1* is a commonly used fibrosis marker and is implicated in ECM remodeling. It is elevated during fibrotic conditions, such as cardiac fibrosis, via its role in myofibroblast differentiation [27]. While minimal *col6a1* expression was detected in wild-type siblings (Figure 3F), we observed patches of *col6a1* expression around the outer layer of both the atrium and ventricle of adult *tnnt2a*^+/RK94del^ zebrafish (*n* = 6) (Figure 3G, arrowheads). Next, we stained for collagen and fibrin using acid fuchsin orange G (AFOG) staining, where we observed increased collagen levels (in blue) and fibrin deposition (in red) within and around the atrium of *tnnt2a*^+/RK94del^, unlike their wild-type siblings (*n* = 6) (Figure 3H,I, arrowhead). We then stained for fibroblast marker, periostin (*postnb*), and we observed an increased expression in *tnnt2a*^+/RK94del^ fish, which overlapped with the areas of elevated *col6a1* expression (*n* = 6) (Figure 3J,K, arrowheads). Taken together, these results indicate a moderate increase in fibrosis, mostly in the outer layer of the atrium in *tnnt2a*^+/RK94del^ fish.

### 3.5. Embryonic tnnt2a^+/RK94del^ fish Show Decreased Heart Rate Accompanied by Changes in Contractile and Hemodynamic Parameters

**Figure 4:**
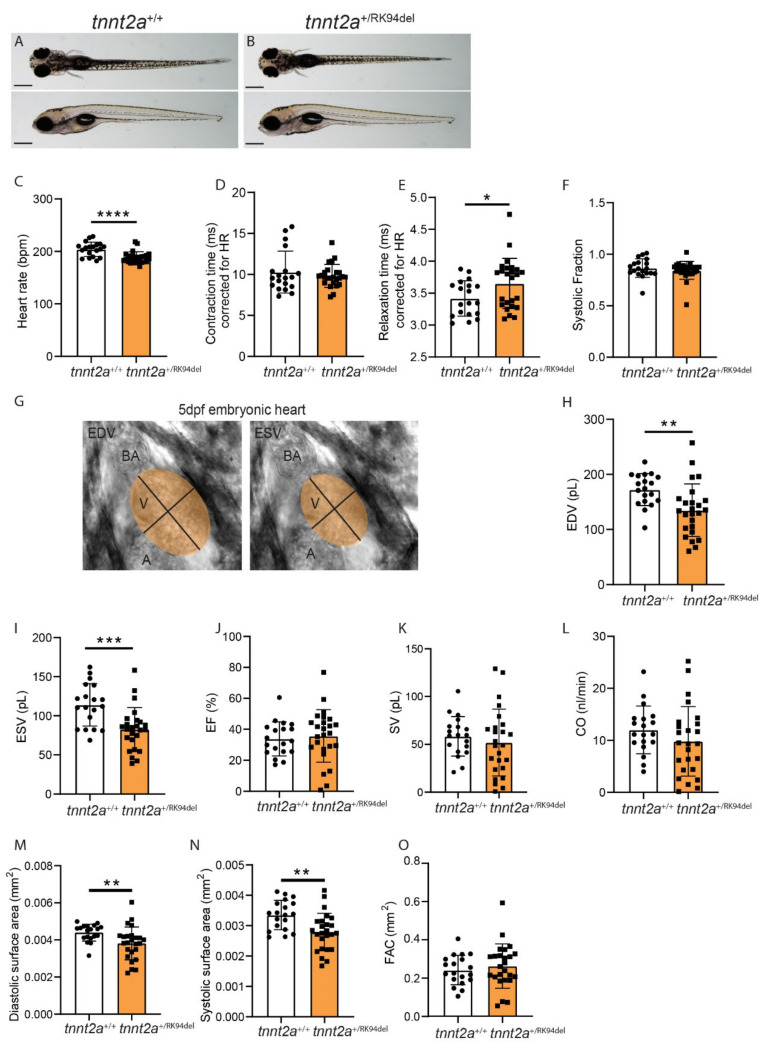
Embryos of *tnnt2a*^+/RK94del^ show decreased heart rate accompanied by decreased ventricular diastolic and systolic volumes, as well as increased relaxation time. Side view and top view of 5 dpf (**A**) *tnnt2a*^+/+^ and (**B**) *tnnt2a*^+/RK94del^ embryos. (**C**–**F**) Bar graph of contractility parameters, including heart rate, contraction time, relaxation time, and systolic fraction for *tnnt2a*^+/+^ and *tnnt2a*^+/RK94del^. (**G**) For hemodynamic parameters, we used ImageJ to fit an ellipse over the ventricle in every recording, both at end-diastole (left) and end-systole (right). (**H**–**L**) Bar graphs of hemodynamic parameters, including end-diastolic volume (EDV), end-systolic volume (ESV), ejection fraction (EF), stroke volume (SV), and cardiac output (CO). (**M**–**O**) Bar graphs of surface area parameters: diastolic surface area, systolic diastolic surface area, and fractional area change (FAC). Scale bar: 100 µm. Statistics: mean ± SEM, * *p* ≤ 0.05, ** *p* ≤ 0.01, *** *p* ≤ 0.001, **** *p* ≤ 0.0001, *tnnt2a*^+/+^ *n* = 19, *tnnt2a*^+/RK94del^ *n* = 24, unpaired Students *t*-test.

Structural changes were detected early on in the developmental stages of the *tnnt2a*^+/RK94del^ hearts, which suggests that very early functional defects may underlie the cardiac remodeling in these mutants. We demonstrated a consistent enlargement of the atrium and a reduction of the ventricle in *tnnt2a*^+/RK94del^ hearts, which appears similar to ventricular dysfunction with atrial compensation [28]. To investigate the role of ventricular dysfunction, we measured early heart rhythm and ventricular contractility in 5 dpf larvae, using brightfield high-speed video recording combined with image analysis. The high-speed video recording was performed on *tnnt2a*^+/RK94del^ embryos without an apparent phenotype and on their wild-type siblings (Figure 4A,B). Image analysis revealed that the heart rate of *tnnt2a*^+/RK94del^ embryos was significantly decreased compared to wild-type siblings (Figure 4C). Next, we measured different contractile parameters to observe the ability of the ventricle to contract and relax. Contraction time (time from maximum relaxation to maximum contraction), relaxation time (time from maximum contraction to maximum relaxation), and systolic fraction (the fraction of time the heart is in systole) were measured and corrected for heart rate. We observed a prolonged relaxation time in the ventricle of *tnnt2a*^+/RK94del^ embryos, indicating that it takes longer before cardiomyocytes return to their uncontracted state (Figure 4E). Contraction time and systolic fraction were not affected (Figure 4D,F). Next, we measured hemodynamic parameters to identify the effect of RK94del mutation on cardiac pump function. An ellipse shape was fitted on the ventricle of brightfield high-speed recordings of embryonic zebrafish hearts (Figure 4G). Using established mathematical formulas, we measured the volume and surface area of each ventricle during diastole and systole. A decrease in end-diastolic volume (EDV) and end-systolic volume (ESV) was observed in *tnnt2a*^+/RK94del^ embryos (Figure 4H,I). Ejection fraction (EF), stroke volume (SV), and cardiac output (CO) remained unaffected (Figure 4J–L). Surface areas were also affected, where there was a significant decrease in systolic and diastolic surface areas, while fractional area change (FAC) remained unaffected (Figure 4M–O). The results shown here demonstrate that seemingly healthy *tnnt2a*^+/RK94del^ embryos already display minor changes in heart rhythm and contractility. These changes likely give rise to the remodeling at adult stages.

### 3.6. The tnnt2a^+/RK94del^ Mutation Causes Impaired Calcium Dynamics in Embryos

**Figure 5:**
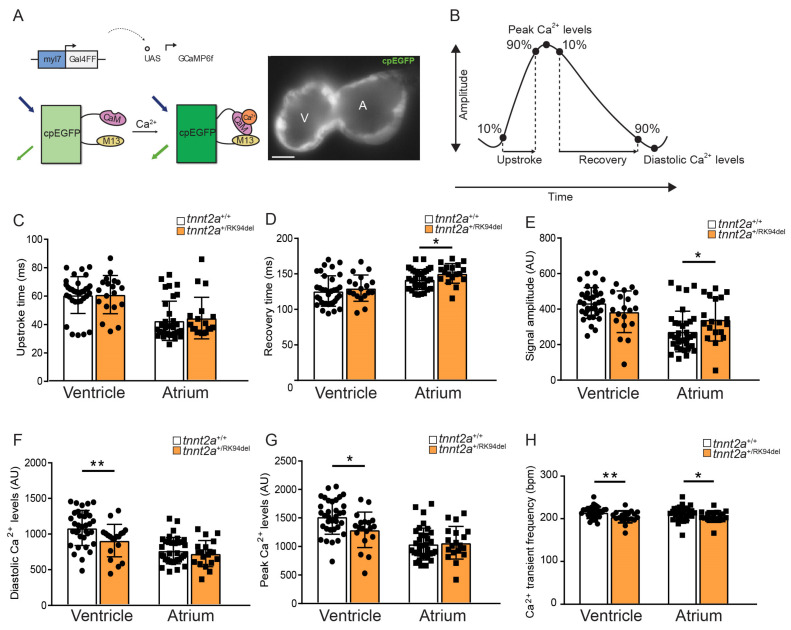
*tnnt2a*^+/RK94del^ mutation causes intracellular calcium dysregulation in zebrafish embryos. (**A**) DNA construct and sensor dynamics of GCaMP6f (left panel). GCaMP6f was placed under the control of the *myl7* promoter to restrict its expression to the heart, where the *Gal4FF-UAS* system amplifies its expression. GCaMP6f consists of a circularly permutated enhanced green fluorescence protein (cpEGFP) fused to calmodulin (CaM) and the M13 peptide. Upon the increase of intracellular calcium (Ca^2+^), CaM binds to M13, causing increased brightness of cpEGFP. This is visualized using a high-speed epifluorescence microscope, where movies of 5 dpf non-contracting GCaMP6f embryonic hearts were recorded (left panel). (**B**) Schematic representation of Ca^2+^ transient parameters examined from the recorded GCaMP6f signals. (**C**–**G**) Bar graphs of the different Ca^2+^ transient parameters for *tnnt2a*^+/+^ and *tnnt2a*^+/RK94del^, including upstroke time, recovery time, signal amplitude, diastolic Ca^2+^ levels, and peak Ca^2+^ levels. (**H**) Frequency of Ca^2+^ transients *tnnt2a*^+/+^ and *tnnt2a*^+/RK94del^. AU: arbitrary unit. Statistics: mean ± SEM, * *p* ≤ 0.05, ** *p* ≤ 0.01, *tnnt2a*^+/+^ *n* = 34, *tnnt2a*^+/RK94del^ *n* = 19, unpaired Students *t*-test. A: atrium, V: ventricle.

While isolating ventricular cardiomyocytes to observe potential structural and functional changes, we observed that cells from *tnnt2a*^+/RK94del^ fish were highly sensitive and always died during the process of isolation, where wild-type cells remained intact. Since cardiomyopathies progress over a longer time span due to a spectrum of defects, including impaired intracellular Ca^2+^ handling, we investigated whether we could observe Ca^2+^ dysfunction well before the end-stages of RK94del remodeling. *tnnt2a*^+/RK94del^ fish were crossed with a *tg* (*myl7*:Gal4FF *UAS*:GCaMP6f) line, expressing a cytosolic cardiac Ca^2+^ sensor [25]. Analysis of GCaMP6f (cpEGFP) signal intensity over time allows the examination of Ca^2+^ transient amplitudes, the speed of intracellular Ca^2+^ release, and the speed of Ca^2+^ reuptake/clearance, as shown earlier [25] (Figure 5A,B). We observed changes in Ca^2+^ parameters in both the ventricle and atrium of 5 dpf *tnnt2a*^+/RK94del^ embryos. No significant differences were observed in Ca^2+^ transient upstroke time between wild-type siblings and *tnnt2a*^+/RK94del^ in both heart chambers (Figure 5C), indicating that the speed of cytosolic Ca^2+^ influx is unaffected in both heart chambers. Ca^2+^ recovery time was significantly prolonged, indicating slower clearance of Ca^2+^ during diastole, and signal amplitudes were significantly increased in the atrium of *tnnt2a*^+/RK94del^ embryos (Figure 5D,E). Atrial diastolic Ca^2+^ levels and peak Ca^2+^ levels were not significantly affected in *tnnt2a*^+/RK94del^ compared to wild-type siblings (Figure 5F,G); however, a significant decrease in both parameters was detected in the ventricle of the *tnnt2a*^+/RK94del^ embryos. This suggests that the ventricle of *tnnt2a*^+/RK94del^ embryos has a reduced amount of cytosolic Ca^2+^ during contraction and relaxation compared to wild-type siblings. Ca^2+^ transient frequency was significantly decreased in both the atrium and ventricle of *tnnt2a*^+/RK94del^, which is likely linked to the decrease in heart rate that we have shown earlier (Figure 5H and Figure 4C). Overall, the atrium of *tnnt2a*^+/RK94del^ embryos shows an increase in Ca^2+^ amplitude and prolonged recovery time, while the ventricle has lower cytosolic Ca^2+^ levels during contraction and relaxation. Therefore, these findings imply that the Ca^2+^ dynamics within the hearts of *tnnt2a*^+/RK94del^ fish are affected as early as 5 dpf.

## 4. Discussion and Conclusions

Previously characterized *sih/tnnt2* mutant alleles in zebrafish result in either no protein or a severely truncated protein only 11-amino-acids-long [20]. In this study, we targeted a location within the *tnnt2a* gene that is well known in human patients as the mutational “hotspot” for cardiomyopathies [9]. This location is within the TNT1 region of the protein, which is involved in the binding of cardiac TnT to Tm and is essential for the function of the TnT protein. The TNT1 region is a highly helical domain that stabilizes the TNT1-Tm interaction and increases the affinity of Tm to actin [29,30,31]. Earlier work showed that mutations in the region between amino acids 92–110 of *TNNT2* impairs Tm binding or the ability of TnT to enhance tropomyosin-actin binding, leading to improper muscle contraction [31,32]. Flexibility of the TNT1 domain is important in allowing the efficient binding to Tm. Mutations in TNT1 that alter its flexibility lead to changes in Ca^2+^ activation in the thin filaments and propagation of the Ca^2+^ signal [33]. The regulatory element of Ca^2+^ is partially retained in the TNT2 domain of TnT; however, for regulation of contractility, Ca^2+^ needs Tm-TNT1 binding to propagate its signal [29]. The changes in TNT1 flexibility promote Ca^2+^ alterations, which have been shown to correspond to Ca^2+^ sensitivity, where increased flexibility leads to increased Ca^2+^ sensitivity [33,34,35]. Indeed, force measurements on detergent-skinned fiber bundles from transgenic mouse cardiomyocytes with mutations in TNT1 found in human cardiomyopathy patients (e.g., R92Q, I79N, and F110I) display increased Ca^2+^ sensitivity [36,37,38,39,40,41]. The observed early defects in Ca^2+^ dynamics and contractile dynamics presented in our study may very well be linked to changes in flexibility of TNT1 due to the deletion of Arginine and Lysine in the mutational hotspot of TnT, leading to inefficient propagation of Ca^2+^ signal to Tm.

The troponin complex is an important regulator of Ca^2+^ dynamics as TnC typically binds 50% of the Ca^2+^ that is released from the SR [42]. An increase in Ca^2+^ sensitivity of the troponin complex, by an increase in Ca^2+^ affinity of TnC, is predicted to result in a decrease in peak [Ca^2+^] [40,43]. This is consistent with our observation of decreased peak Ca^2+^ levels in the ventricles of *tnnt2a*^+/RK94del^ embryos measured with the GCaMP6 sensor. A limitation of the GCaMP sensor is that it does not allow measurement of exact [Ca^2+^]. This would require measurements on isolated cells, which is hampered by the difficulty to isolate intact cardiomyocytes from embryos and from adult *tnnt2a*^+/RK94del^ hearts. A higher affinity of the troponin complex for Ca^2+^ would also result in a slower release of Ca^2+^ into the cytoplasm and a slower clearance from the cell, which translates to a slower Ca^2+^ recovery and prolonged relaxation time. Indeed, we observed prolonged relaxation times in *tnnt2a*^+/RK94del^ embryos. In addition, a decrease in diastolic Ca^2+^ levels in the ventricle of *tnnt2a*^+/RK94del^ embryos was detected, suggesting that more Ca^2+^ was bound to troponin when TnT-RK94del is present in the complex. More Ca^2+^ bound to troponin at diastole is also consistent with the observed reduced EDV of the ventricles in *tnnt2a*^+/RK94del^ embryos. Taken together, the reduced EDV and ESV combined with the reduction in cytoplasmic Ca^2+^ levels at diastole and systole are consistent with an increase in Ca^2+^ sensitivity of the troponin complex due to the TnT-RK94del mutation.

Structural remodeling observed in *tnnt2a*^+/RK94del^ adult zebrafish is characterized by a reduced ventricular size together with a severely enlarged atrium. The model presented here appears similar to heart failure with preserved EF (HFpEF), as we observed diastolic dysfunction with atrial enlargement while maintaining EF level, a classic appearance of HFpEF [44,45]. The atrium, however, demonstrates increased myocardial stress, collagen deposition, and fibrosis during later stages, causing loss of elasticity and eventual heart failure. Interestingly, transgenic mice expressing a truncated form of Troponin T or mutated form of Troponin T (R92Q mutation) show a similar diastolic dysfunction combined with a decrease in ventricle size and a larger hypertrophic atrium [46,47]. A similar diastolic dysfunction combined with enlargement of the atria has been documented in patients with HCM, including familial cases with mutations in *TNNT2* affecting the TNT1 domain [11,12,28,48]. Left atrium dilation is associated with ventricular diastolic dysfunction, which comes as a result of abnormal left ventricular relaxation and changes in actin-myosin interaction [49]. The diastolic dysfunction causes the left atrium pressure to continually increase in order to maintain a proper filling, leading to atrial wall tension and causing dilation and stretching of the atrial myocardium.

In summary, we suggest that the RK94del mutation in TnT affects the ability of Tm to bind TnT, leading to changes in Ca^2+^ dynamics, increased Ca^2+^ sensitivity, and contractility defects. We showed that the ventricle displays a form of HFpEF, where diastolic function is dysregulated while EF is maintained. The atrium, in this case, is enlarged to relieve the pressure and promote the proper ventricular filling. At a point where the atrium is unable to efficiently maintain the heart function, remodeling, involving increased collagen deposits and fibrosis, progresses ultimately leading to heart failure. The *tnnt2a*^+/RK94del^ fish model we described here may help identify potential therapeutics in the future, which have the potential to reduce Ca^2+^ sensitivity and prevent cardiac remodeling in patients. As zebrafish have a highly conserved drug metabolism, physiology, and pharmacology, and the larvae are small enough to grow in a 96-well plate, they present an ideal model for drug discovery [50,51].

## Data Availability

Not applicable.

## Supplementary Materials

The following are available online at https://www.mdpi.com/article/10.3390/jcdd8040046/s1, Figure S1: Sequences for primers and oligonucleotides used in this study, Figure S2: Heart rate from a brightfield high-speed video imaging of tnnt2a+/hu11260 at 5 dpf, Figure S3: Representative embryonic pictures of the different genotypes at 3 dpf and 5 dpf embryos.
