# Supplementary material for: A Heterozygous Mutation in Cardiac Troponin T Promotes Ca2+ Dysregulation and Adult Cardiomyopathy in Zebrafish
Source: J Cardiovasc Dev Dis. 2021 Apr 20;8(4):46. doi: 10.3390/jcdd8040046 (PMC8072640; doi:10.3390/jcdd8040046)
Supplement: Supplementary file 1 [file jcdd-08-00046-s001.zip › jcdd-1156166-supplementary.pdf]

Supplementary information

A. Sequencing primers

Primer name	Sequence	Size (bp)
Tnnt2a_ZF_Fw	CCGTTTGCTTTGTGGGTTTTGTCA	301
Tnnt2a_ZF_Rv	TGTTTTCGCGAATTTACCCCACTG	301

B. sgRNA

Oligo name	Sequence
Tnnt2a_ZF_Oligo 1	TAGGTCCTTCTCCATGCGCTTA

Supplementary Figure 1: Sequences for primers and oligonucleotides used in this study. (A) Sequences of PCR primers used for sequencing. **(B)** Sequence of sgRNAs used for targeting of the Cas9 nuclease to the specific site(s) of mutation(s).

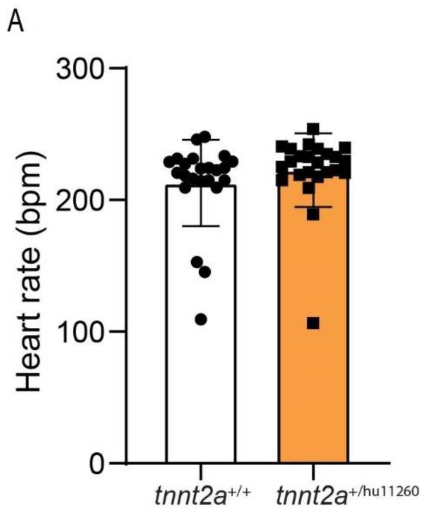

Supplementary Figure 2. Heart rate from a brightfield high-speed video imaging of *tnnt2a* $^{+/hu11260}$ at 5 dpf. (A) Bar graph of heart rate for *tnnt2a*$^{+/+}$and *tnnt2a*$^{+/hu11260}$. Statistics: mean ± SEM, n.s. p>0.05, *tnnt2a*$^{+/+}$ n=23, *tnnt2a*$^{+/hu11260}$ n=24, unpaired Students t-test.

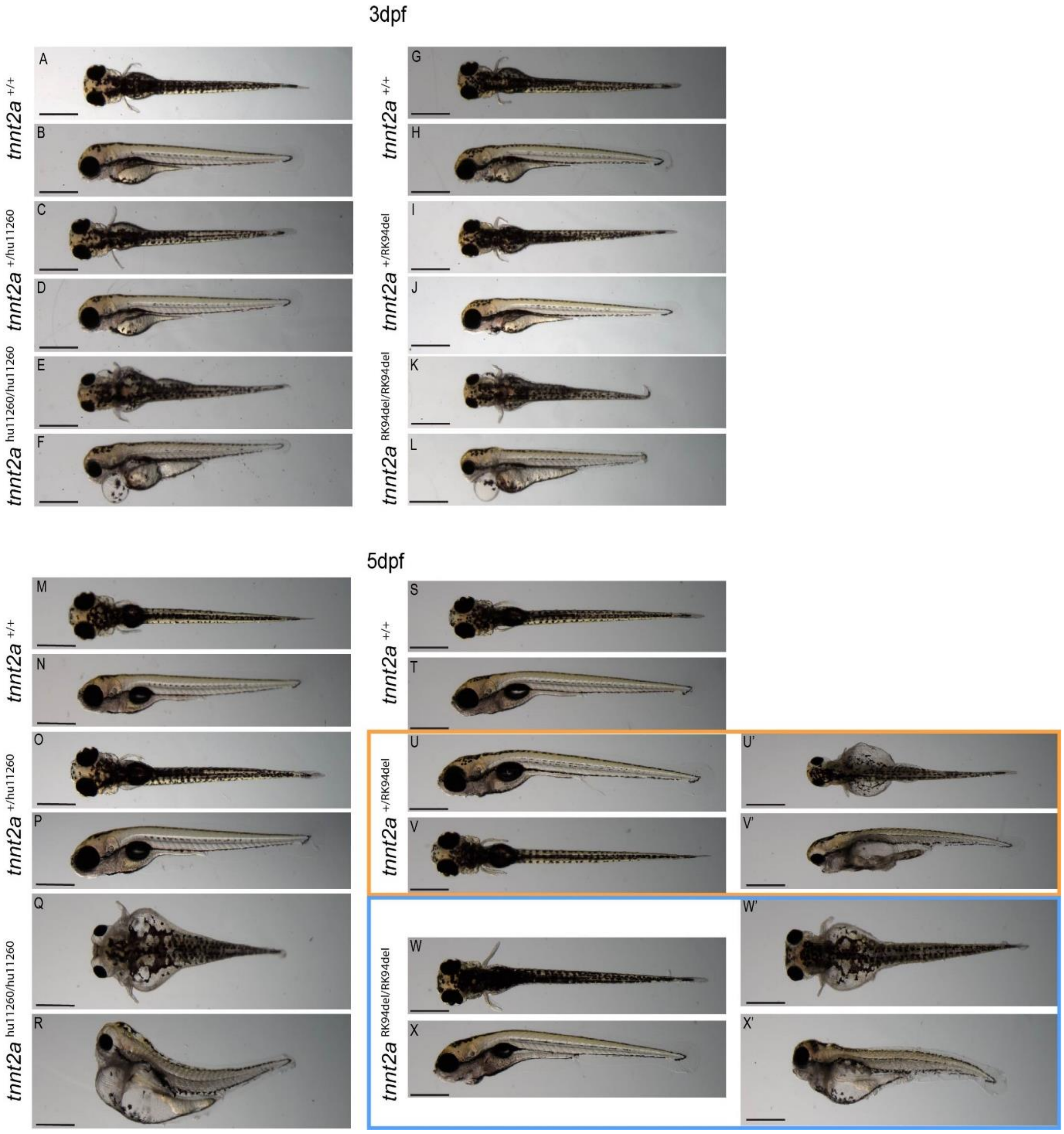

Supplementary Figure 3: Representative embryonic pictures of the different genotypes at 3 dpf and 5 dpf embryos. (**A-F**) Images of *tnnt2a*$^{+/+}$, *tnnt2a*$^{+/hu11260}$, *tnnt2a*$^{hu11260/hu11260}$ at 3 dpf. (**G-L**) Images of *tnnt2a*$^{+/+}$, *tnnt2a*$^{+/RK94del}$, *tnnt2a*$^{RK94del/RK94del}$ at 3 dpf. (**M-R**) Images of *tnnt2a*$^{+/+}$, *tnnt2a*$^{+/hu11260}$, *tnnt2a*$^{hu11260/hu11260}$ at 5 dpf. (**S-X**) Images of *tnnt2a*$^{+/+}$, *tnnt2a*$^{+/RK94del}$, *tnnt2a*$^{RK94del/RK94del}$ at 5 dpf. (**U',V',W',X'**) Representative images of the phenotype variations of *tnnt2a*$^{+/RK94del}$ and *tnnt2a*$^{RK94del/RK94del}$ at 5 dpf. Scale bar: 100 µm.
